# Deep molecular responses for treatment‐free remission in chronic myeloid leukemia

**DOI:** 10.1002/cam4.801

**Published:** 2016-07-01

**Authors:** Stéphanie Dulucq, Francois‐Xavier Mahon

**Affiliations:** ^1^ Laboratoire d'HématologieCentre Hospitalier Universitaire de BordeauxBordeauxFrance; ^2^ Bergonié Cancer InstituteINSERM Unit 916University of BordeauxBordeauxFrance

**Keywords:** Chronic myeloid leukemia, deep molecular response, treatment‐free remission, tyrosine kinase inhibitors

## Abstract

Several clinical trials have demonstrated that some patients with chronic myeloid leukemia in chronic phase (CML‐CP) who achieve sustained deep molecular responses on tyrosine kinase inhibitor (TKI) therapy can safely suspend therapy and attempt treatment‐free remission (TFR). Many TFR studies to date have enrolled imatinib‐treated patients; however, the feasibility of TFR following nilotinib or dasatinib has also been demonstrated. In this review, we discuss available data from TFR trials and what these data reveal about the molecular biology of TFR. With an increasing number of ongoing TFR clinical trials, TFR may become an achievable goal for patients with CML‐CP.

## Introduction

Treatment outcomes and survival rates for patients with chronic myeloid leukemia in chronic phase (CML‐CP) have improved substantially since the introduction of imatinib 1, 2 and the subsequent development of second‐generation tyrosine kinase inhibitors (TKIs) 3, 4, 5. The success of TKI therapy has evolved CML into a model for targeted cancer therapy; now high rates of deep molecular response and reports of patients successfully maintaining treatment‐free remission (TFR) in clinical trials are turning CML into a model for addressing the concept of curability. Published data from several clinical trials have demonstrated the safety and feasibility of TFR for some patients who respond well to TKI therapy 6, 7, 8, 9, 10, 11, 12, 13, 14, 15, 16, 17. Although current CML treatment recommendations from the National Comprehensive Cancer Network (NCCN) and European Leukemia Net (ELN) call for patients to remain on TKI therapy indefinitely, both the NCCN and ELN note that some patients may be able to attempt TFR within the context of a clinical trial and under physician supervision 18, 19.

TFR may be an attractive goal for patients and physicians for a number of reasons. Although patients who respond well to TKI therapy have survival rates similar to those observed in the general population 20, the impact of long‐term TKI therapy on patients’ lives must be considered. TKI‐associated adverse events (AEs) that affect daily living are observed in approximately 30% of patients, and such AEs have been shown to impact quality of life during long‐term TKI therapy 21, 22. This may be especially true for younger patients. Relative to age‐matched peers from the general population, younger patients (i.e., those aged 18–39 years) have been shown to experience greater quality‐of‐life impact from CML and TKI therapy than older patients 23. If suspension of TKI therapy leads to resolution of TKI‐associated AEs, achievement of successful TFR may result in substantial quality‐of‐life improvements for these patients. In addition, there is evidence of growth impairment in pediatric patients with CML treated with imatinib 24; although TFR studies to date have focused on adult patients 6, 7, 8, 9, 10, 11, 12, 13, 14, 15, 16, 17, there may also be significant long‐term benefits of TFR for pediatric patients. Furthermore, it is recommended that patients do not become pregnant while receiving any TKI due to potential embryotoxicity 25, 26, 27, 28, 29. In patients with CML who wish to become pregnant, TFR may represent a safer setting for pregnancy. TFR may also have economic benefits 10, 30, 31, 32. With a median follow‐up of 50 months after suspension of imatinib in the Stop Imatinib (STIM1) TFR clinical trial, the total estimated saving for the 100 enrolled patients was 5.5 million euros 32. Results from several surveys of TKI‐treated patients with CML‐CP have reported that quality‐of‐life improvements (e.g., relief from AEs) and financial benefits were among the most frequently reported potential motivators for attempting TFR 33, 34, 35, 36. In addition, some surveyed patients stated that expectations of positive emotional impacts from successful TFR would motivate them to attempt TFR 33.

Results from clinical trials and case studies suggest certain requirements for successful TFR. Although many patients with sustained deep molecular responses (typically undetectable minimal residual disease [UMRD]) can achieve successful TFR 6, 7, 8, 9, 10, 11, 12, 13, 14, 15, 16, 17, those who attempted TFR without deep molecular responses rapidly required reinitiation of therapy 37, 38. After achievement of a deep molecular response, maintenance of that response for some period with continued TKI therapy appears to be important for successful TFR 9, although the necessary duration of sustained response is not known. Other clinical and biological factors required for successful TFR are being investigated in ongoing trials 7, 9, 12, 15, 16, 17, 39, 40, 41. In this review, we discuss the clinical significance of deep molecular responses for patients with CML‐CP, results from clinical trials of TFR, and clinical and biological factors that may predict TFR.

## Deep Molecular Responses in CML

For patients with CML‐CP, the deepest levels of response designated as treatment goals by the NCCN and ELN are complete cytogenetic response (CCyR; 0% Philadelphia chromosome [Ph]+ metaphases) and major molecular response (MMR; *BCR‐ABL1* transcript ratio ≤0.1% on the International Scale [IS; *BCR‐ABL1* ^IS^]), respectively 18, 19. Over time, many patients achieve deeper molecular responses, such as molecular response 4 (MR^4^; *BCR‐ABL1* ^IS^ ≤0.01%) and molecular response 4.5 (MR^4.5^; *BCR‐ABL1* ^IS^ ≤0.0032%), and some patients achieve responses beyond the limit of detection of the assays used. This level of response is often referred to as a complete molecular response (CMR) or UMRD; however, because assay sensitivity varies between studies, laboratories, and samples, the definitions of CMR and UMRD are not standardized and must be detailed in any report using these terms 18.

### Recommendations for measuring deep molecular response

To address the need for improved standardization between laboratories in the analysis and reporting of deep molecular responses, the European Treatment and Outcome Study (EUTOS) group developed detailed technical laboratory recommendations for measuring and scoring deep molecular responses 42. These recommendations are critically important for TFR studies, in which deep molecular responses must be routinely monitored with high accuracy, precision, and reproducibility. First, laboratories must quantify, typically using real‐time quantitative polymerase chain reaction (RQ‐PCR), the ratio of *BCR‐ABL1* transcripts against those of a control gene such as *ABL1*,* GUSB*, or *BCR* 42. RQ‐PCR results must be converted to the IS using a laboratory‐specific conversion factor or calibrated reagents or kits 42, 43, 44, 45, 46. EUTOS recommends that both *BCR‐ABL1* and the control gene be evaluated in ≥2 replicates from the same sample and that the same volume of complementary DNA (cDNA) be used for *BCR‐ABL1* and control gene transcript estimation 42, 47. The sensitivity of the assay can be enhanced by increasing the amount of sample being analyzed, but calculation adjustments must be made if the number of replicates differs between *BCR‐ABL1* and the control gene 42.

A minimum number of control gene transcripts are required for each replicate and for the sum of all replicates to determine the level of molecular response in samples with either detectable or undetectable *BCR‐ABL1* transcript 42. To be considered evaluable, each replicate should contain ≥10,000 *ABL1* transcripts or ≥24,000 *GUSB* transcripts 42, 47. A minimum total of 10,000 *ABL1* transcripts or 24,000 *GUSB* transcripts in all replicates are required to assess MR^4^ 42. To assess MR^4.5^ and molecular response 5 (MR^5^; *BCR‐ABL1* ^IS^ ≤0.001%), a minimum total of 32,000 *ABL1* or 77,000 *GUSB* transcripts and 100,000 *ABL1* or 240,000 *GUSB* transcripts, respectively, in all replicates are required 42. For laboratories using *BCR* as a reference gene, further work is needed to determine the minimum number of transcripts necessary to evaluate each level of response 42.

Specific precautions are recommended by EUTOS to minimize the risk of false‐positive or false‐negative samples 42. To minimize false positivity, EUTOS recommends a positive cutoff equal to quantification cycle (Cq) of intercept +1 (i.e., samples with a Cq > intercept +1 should be considered negative) and the use of negative controls (e.g., no‐template control wells and reagent blanks) 42. To minimize false negativity, a sample should be considered UMRD only if all sample replicates show no *BCR‐ABL1* 42. It is also important for laboratories to determine and optimize the assay limit of detection (defined by EUTOS as the lowest concentration of *BCR‐ABL1* detectable with 95% confidence 42) to improve the precision with which a low *BCR‐ABL1* transcript number can be measured. In a laboratory with a nonoptimized limit of detection, a patient's sample may falsely be scored as deep molecular response due to failure to detect *BCR‐ABL1* 42. The limit of detection can be determined with the use of standardized reference reagent panels or the ERM‐AD623 plasmid 44, 46. Because the lowest theoretical limit of detection based on the Poisson distribution is 3 *BCR‐ABL1* copies per sample, EUTOS recommends that any positive sample be reported as having ≥3 copies 42.

### Clinical significance of deep molecular response

In addition to the potential for successful TFR, multiple groups have demonstrated that achievement of deep molecular responses may be associated with improved long‐term clinical outcomes, including higher rates of survival and avoidance of disease progression 5, 48, 49. In a retrospective study by Falchi et al. 5, patients who achieved undetectable *BCR‐ABL1* (in a standardized assay able to detect 1 *BCR‐ABL1* transcript in 100,000 *ABL1* copies) at any time (eight patients of 215 evaluable) had better 6‐year overall survival probability versus patients with a best response of MMR (8/61 patients, *P* < 0.0001 vs. undetectable *BCR‐ABL1*) and versus those without a molecular response (7/33 patients, *P* < 0.0001 vs. undetectable *BCR‐ABL1*). In CML Study IV, patients with MR^4.5^ at 4 years (*n* = 198) had a higher probability of survival at 8 years versus patients with *BCR‐ABL1* ^IS^ of 0.1–1% (*n* = 55; 8‐year survival, 92% vs. 83%, respectively; *P* = 0.047), and no patient who achieved MR^4.5^ in that analysis progressed to accelerated phase/blast crisis by the data cutoff 48. In a retrospective analysis of 180 patients with CML‐CP treated with frontline imatinib, Etienne et al. 49 found that those who achieved confirmed CMR (defined in that study as MR^4.5^ with undetectable *BCR‐ABL1* transcripts) at any time (*n* = 65) had higher event‐free survival (95.2%) versus those who achieved CCyR and MMR, but not CMR (*n* = 92; 64.7%; *P* = 0.00124 vs. patients with CMR), and versus those who achieved CCyR, but not MMR (*n* = 23; 27.7%; *P* < 0.0001 vs. patients with CMR). A landmark analysis of outcomes according to response level at 18 months detected a trend (*P* = 0.11) for higher rates of event‐free survival among patients with CMR (*n* = 19) versus patients with CCyR and MMR, but not CMR (*n* = 101), and versus those with CCyR, but neither MMR nor CMR (*n* = 72) 49.

With a growing body of data linking deep molecular responses to good patient outcomes, the importance of increasing the proportion of patients who can achieve such responses is becoming clear. Several studies have demonstrated that more patients are able to achieve deep molecular responses with the second‐generation TKIs nilotinib and dasatinib than with imatinib. For example, in the Evaluating Nilotinib Efficacy and Safety in Clinical Trials–Newly Diagnosed Patients (ENESTnd) study comparing frontline nilotinib versus imatinib for patients with CML‐CP, 56% (nominal *P* < 0.0001 vs. imatinib) of patients in the nilotinib 300 mg twice‐daily arm, 55% (nominal *P* < 0.0001 vs. imatinib) of patients in the nilotinib 400 mg twice‐daily arm, and 33% of patients in the imatinib arm achieved MR^4.5^ by 6 years 50. In the Dasatinib versus Imatinib Study in Treatment‐Naive CML Patients (DASISION) study comparing frontline dasatinib versus imatinib, 42% of patients in the dasatinib arm versus 33% of patients in the imatinib arm achieved MR^4.5^ by 5 years (*P* = 0.025) 3. In a retrospective analysis of 483 consecutive patients with CML‐CP treated with imatinib, nilotinib, or dasatinib at a single institution, Falchi et al. 5 reported that 25% (11 of 44) of those treated with imatinib 400 mg/day, 33% (48 of 147) of those treated with imatinib 800 mg/day, 35% (17 of 48) of those treated with nilotinib, and 34% (19 of 56) of those treated with dasatinib achieved MR^4.5^ by 3 years. In addition, patients with detectable *BCR‐ABL1* on long‐term imatinib can achieve deeper molecular response by switching to nilotinib 51. In the ENEST–Complete Molecular Response (ENESTcmr) study of patients with minimal residual disease after ≥2 years on imatinib, patients randomized to switch to nilotinib achieved higher rates of MR^4.5^ and undetectable *BCR‐ABL1* versus those randomized to remain on imatinib 51.

## Results From Clinical Trials of TFR

Using different study designs (Table 1) and triggers to reinitiate TKI therapy (Table 2), several clinical studies have demonstrated the feasibility of TFR for some patients with CML. Together, results from these studies are providing initial guidance on appropriate criteria for identifying patients who may be able to achieve TFR. STIM1 was among the first large, prospective studies of TFR in patients with deep molecular responses 6, 52. STIM1 enrolled patients who had received imatinib therapy for ≥3 years and had maintained undetectable *BCR‐ABL1* (in five assessments, confirmed at screening in a central laboratory with sensitivity to >4.7‐log reduction in *BCR‐ABL1* transcripts) for ≥2 years while receiving imatinib. Reinitiation of TKI therapy was triggered by *BCR‐ABL1* positivity in two consecutive assessments with a *BCR‐ABL1/ABL1* ratio of ≥10^−5^ and a 1‐log increase in the second assessment relative to the first (i.e., loss of CMR) 6, 52. With a median follow‐up of 30 months in STIM1, molecular relapse was reported in 61 of 100 patients, and the probability of sustained CMR was 39% (95% CI, 29–48%) at both 24 and 36 months. Most relapses (58 of 61) occurred during the first 7 months after suspension of imatinib, and three late relapses occurred at months 19, 20, and 22 52. With longer follow‐up (median of 65 months), no additional molecular relapses were reported 16.

**Table 1:** Molecular biology criteria in TFR studies

Study	Evaluation to determine eligibility for attempting TFR	Molecular monitoring during TFR
STIM1 6	CMR sustained for ≥2 years, with UMRD in five assessments and confirmed at screening in a central laboratory with sensitivity to >4.7‐log reduction in *BCR‐ABL1* transcript levels	Monthly for the first 12 months, every 2 months in year 2, and every 3 months thereafter
TWISTER 7	UMRD sustained for ≥2 years, with monitoring at local laboratories and confirmed at screening in a central laboratory with ≥4.5‐log sensitivity	Monthly for 12 months, every 2 months in year 2, and every 3 months thereafter
A‐STIM 12	Confirmed CMR—either stable (UMRD in all assessments) or unstable (occasional detectable *BCR‐ABL1* ^IS^ <0.1%)—for ≥2 years, with assessments every 6 months at local standardized laboratories and ≥40,000 amplified copies of *ABL1* in each assessment	Monthly for the first 12 months, every 2 months in year 2, and every 3 months thereafter
KIDS 9	UMRD sustained for ≥2 years, with duplicate analyses at >6 time points and a screening assessment performed in a central laboratory with ≥4.5‐log sensitivity with nested RT‐PCR and duplicate RQ‐PCR assessments	Monthly for the first 6 months, every 2 months through month 12, and every 3 months thereafter
EURO‐SKI 13, 55	MR^4^ in three consecutive assessments over the course of >12 months, with final confirmation of MR^4^ performed in a standardized laboratory	Every 4 to 6 weeks for the first year and every 3 months in year 2 and 3
STOP 2G‐TKI 14	UMRD (undetectable MR^4.5^) for ≥24 months	Monthly for the first 12 months, every 2–3 months in year 2, and every 3–6 months thereafter
DADI 15	Deep molecular response sustained for ≥1 year, with assessments every 3 months at a central standardized laboratory (assay sensitivity, 10 copies in 200 ng total RNA; corresponding to *BCR‐ABL1* ^IS^ 0.0069% or MR^4^ [*BCR‐ABL1* ^IS^ ≤0.01% or undetectable disease in cDNA with >10,000 *ABL1* transcripts])	Monthly for the first 12 months, every 3 months in year 2, and every 6 months in year 3
ISAV 54	CMR sustained for ≥18 months, with ≥3 RQ‐PCR tests performed locally	Monthly for the first 6 months, then every 2 months for 36 months

A‐STIM, According to STIM; CMR, complete molecular response; cDNA, complementary DNA; DADI, Dasatinib Discontinuation; EURO‐SKI, European Stop Tyrosine Kinase Inhibitor; ISAV, Imatinib Suspension and Validation; IS, International Scale; KIDS, Korean Imatinib Discontinuation Study; MMR, major molecular response (*BCR‐ABL1* ^IS^ ≤ 0.1%); MR^4^, *BCR‐ABL1* ^IS^ ≤ 0.01%; MR^4.5^, *BCR‐ABL1* ^IS^ ≤ 0.0032%; RQ‐PCR, real‐time quantitative polymerase chain reaction; RT‐PCR, reverse transcriptase polymerase chain reaction; STIM, Stop Imatinib; STOP 2G‐TKI, Stop Second‐Generation Tyrosine Kinase Inhibitor; TFR, treatment‐free remission; UMRD, undetectable minimal residual disease.

**Table 2:** Molecular response trigger for reinitiation of TKI therapy according to *BCR‐ABL1* ^IS^ transcript levels in selected TFR studies

Study	Trigger for reinitiation of therapy	
	Detectable transcripts	Loss of MMR
STIM1 16	Two consecutive assessments (≥1‐log increase between measurements)	Single assessment
STIM2 10	Two consecutive assessments (≥1‐log increase between measurements)	Single assessment
TWISTER 7	Two consecutive assessments	Single assessment
A‐STIM 12		Single assessment
KIDS 9		Two samples within 4 weeks
EURO‐SKI 13, 55		Single assessment
STOP 2G‐TKI 14		Single assessment
DADI 15	Single assessment (*BCR‐ABL1* ^IS^ ≥0.0069% in any assessment)	
ISAV 54	Two consecutive assessments (*BCR‐ABL1* ^IS^ ≥0.1% in one assessment)	

A‐STIM, According to STIM; DADI, Dasatinib Discontinuation; EURO‐SKI, European Stop Tyrosine Kinase Inhibitor; ISAV, Imatinib Suspension and Validation; IS, International Scale; KIDS, Korean Imatinib Discontinuation Study; MMR, major molecular response (*BCR‐ABL1* ^IS^ ≤ 0.1%); STIM, Stop Imatinib; STOP 2G‐TKI, Stop Second‐Generation Tyrosine Kinase Inhibitor; TFR, treatment‐free remission; TKI, tyrosine kinase inhibitor; UMRD, undetectable minimal residual disease.

The results of STIM1 were confirmed in the TWISTER study, which used a similar study design to that of STIM1. TWISTER enrolled patients who had received ≥3 years of imatinib therapy and had maintained UMRD for ≥2 years (UMRD was confirmed prior to enrollment in a central laboratory with ≥4.5‐log assay sensitivity) 7. In TWISTER, reinitiation of TKI therapy was triggered by loss of MMR in any sample or two consecutive assessments with *BCR‐ABL1* positivity 7. With a median follow‐up of 42 months in TWISTER, molecular relapse was reported in 22 of 40 patients; the estimated rate of TFR at 24 months was 47.1% (95% CI, 31.5–62.7%) 7. The majority of relapses in TWISTER (15 of 22) occurred within the first 6 months after suspension of imatinib; no relapses were observed after >27 months of TFR 7.

Several additional studies have confirmed the feasibility of TFR in patients with UMRD on imatinib 8, 10, 11, 53. The “According to STIM” (A‐STIM) study provided new evidence that stable UMRD prior to suspension of therapy is not essential for maintaining TFR and, furthermore, that some patients with low levels of detectable *BCR‐ABL1* after suspension of therapy can maintain TFR without losing MMR 12. In A‐STIM, molecular relapse was defined as loss of MMR. With a median follow‐up of 31 months (range, 8–92 months), 29 of 80 patients (36%) lost MMR and 45 of 80 (56%) lost CMR. Most MMR losses occurred during the first 6 months, with four occurring in months 7–17. In contrast, eight patients lost CMR >6 months after suspension of therapy, including two patients who lost CMR at months 35 and 40 12. At 24 months after suspension of therapy in A‐STIM, the cumulative incidence of molecular relapse was 36% (95% CI, 26–47%); using the STIM1 definition (i.e., loss of CMR), the cumulative incidence of molecular relapse at 24 months in A‐STIM would have been 54% (95% CI, 44–66%) 12. Enrollment criteria for A‐STIM were generally similar to those for STIM1; however, unlike STIM1, A‐STIM enrolled patients with occasional *BCR‐ABL1* positivity prior to suspension of imatinib therapy. In A‐STIM, at 12 months after suspension of imatinib, estimated rates of relapse‐free survival (RFS; i.e., no loss of MMR) were similar for patients who had occasional *BCR‐ABL1* positivity prior to enrollment (RFS, 64%; 95% CI, 48–77%) and for those with stable UMRD prior to enrollment (RFS, 65%; 95% CI, 47–78%) 12. Similar findings were observed in the Korean Imatinib Discontinuation Study (KIDS). KIDS enrolled patients who had received >3 years of imatinib therapy and had ≥2 years of sustained UMRD (confirmed by duplicate assessments at >6 time points) and defined molecular relapse as confirmed loss of MMR 9. Among 90 patients enrolled in KIDS with a minimum follow‐up of 12 months (median follow‐up, 26.6 months; range, 12.6–58.1 months), the probability of sustained MMR at 12 months was 62.2% 9. In the Imatinib Suspension and Validation (ISAV) study, 112 patients with ≥2 years of imatinib therapy with UMRD for ≥18 months (confirmed with ≥3 RQ‐PCR assessments) were enrolled 54. The rate of molecular relapse (two consecutive positive RQ‐PCR samples with ≥1 sample with *BCR‐ABL1/ABL1* >0.1%) was 49.1% at 24 months 54.

The feasibility of TFR following nilotinib or dasatinib has also been demonstrated. The Stop Second‐Generation Tyrosine Kinase Inhibitor (STOP 2G‐TKI) study enrolled patients who had received ≥3 years of TKI therapy, were currently receiving either nilotinib or dasatinib as frontline therapy or following imatinib, and had maintained UMRD (i.e., undetectable MR^4.5^, as assessed in standardized laboratories) for ≥2 years 14. Similar to A‐STIM, reinitiation of TKI therapy in STOP 2G‐TKI was triggered by loss of MMR 14. With a median follow‐up of 32 months (range, 12–56 months) in STOP 2G‐TKI, the estimated rate of TFR (no loss of MMR) at 12 months was 61.4% (95% CI, 48.1–74.6%) 39.

The Japanese Dasatinib Discontinuation (DADI) study evaluated TFR after suspension of dasatinib as second‐line therapy or beyond 15. Sustained deep molecular response for ≥1 year was required prior to suspension of therapy; evaluation of *BCR‐ABL1* levels was performed at a central laboratory standardized to the IS (assay sensitivity, 10 copies in 200 ng total RNA, corresponding to *BCR‐ABL1* ^IS^ 0.0069% or MR^4^ [*BCR‐ABL1* ^IS^ ≤0.01% or undetectable disease in cDNA with >10,000 *ABL1* transcripts]), and molecular relapse was defined as *BCR‐ABL1* ^IS^ ≥0.0069% in any assessment. With a median follow‐up of 20 months (interquartile range, 16.5–24 months), among 63 patients who attempted TFR, deep molecular response was maintained in 30 patients and 33 patients had molecular relapse; the probability of TFR was 49% (95% CI, 36–61%) and 48% (95% CI, 35–59%) at 6 and 12 months, respectively 15. All molecular relapses occurred within 7 months of stopping dasatinib therapy, and upon treatment reinitiation (dasatinib, *n* = 32; nilotinib, *n* = 1) all 33 patients with molecular relapse regained deep molecular response within 6 months and the majority (*n* = 29) did so within 3 months 15.

Preliminary results have also been presented from the European Stop Tyrosine Kinase Inhibitor (EURO‐SKI) study, a large, ongoing trial evaluating TFR in a wider population of patients than those of previous studies. Prior to enrollment in EURO‐SKI, patients must have received TKI therapy for ≥3 years (including frontline therapy, second‐line therapy due to toxicity of frontline therapy, and/or TKI combination therapy) and must have maintained MR^4^ for ≥1 year; molecular relapse was defined as loss of MMR 13, 55. Among the first 200 patients in EURO‐SKI, 61.5% (123 of 200) remained in MMR at 6 months 13; this is comparable to the TFR rate in A‐STIM (at 12 months, 64% [95% CI, 54–75%]) 12 and suggests that patients with sustained MR^4^ may be able to maintain TFR. Several other ongoing studies are evaluating TFR following therapy with nilotinib or dasatinib, TKI dose de‐escalation, or combination therapy with pegylated interferon‐α plus TKI; each has distinct requirements for attempting TFR and distinct triggers for reinitiating therapy (Table 3) 55, 56. Results from these studies will further elucidate this investigational treatment approach.

**Table 3:** Ongoing TFR studies without results reported 55, 56, 66, 67

Study	Treatment prior to suspension of therapy	Study criteria for suspension of therapy	Trigger to reinitiate therapy
Nilo Post‐STIM (NCT01774630)	2 years of nilotinib in patients who failed TFR in STIM1, STIM2, or EURO‐SKI	Stable CMR for 2 years	Confirmed loss of CMR
ENESTop (NCT01698905)	≥3 years of TKI therapy prior to enrollment, including ≥4 weeks of frontline imatinib and ≥2 years of second‐line nilotinib, followed by a 1‐year nilotinib consolidation phase on study	≥1 year MR^4.5^	Loss of MMR or confirmed loss of MR^4^
ENESTpath (NCT01743989)	≥2 years of imatinib followed by 2 or 3 years of nilotinib	MR^4^ (≥1 year or ≥2 years)	Loss of MMR or confirmed loss of MR^4^
ENESTgoal (NCT01744665)	≥1 year of imatinib followed by ≥2 years of nilotinib	≥2 years deep MR	Loss of MMR
ENESTfreedom (NCT01784068)	≥2 years of frontline nilotinib prior to enrollment followed by a 1‐year nilotinib consolidation phase on study	≥1 year MR^4.5^	Loss of MMR
DASFREE (NCT01850004)	≥2 years of dasatinib	≥1 year MR^4.5^, confirmed at screening by a central laboratory	Loss of MMR
Dasatinib Stop (NCT01627132)	Dasatinib	CMR	Loss of CMR
Study for Cure D‐NEWS (NCT01887561)	Dasatinib	CMR	Loss of CMR
TIGER (NCT01657604)	Nilotinib or nilotinib + PEG‐IFNα	Stable MR^4^	Loss of MMR
DESTINY (NCT01804985)	≥3 years of imatinib, nilotinib, or dasatinib followed by 1 year at half‐standard dose	MMR or MR^4^ before dose de‐escalation	Loss of MMR
Imatinib or nilotinib with pegylated interferon‐α 2b in chronic myeloid leukemia (NCT00573378)	≥2 years of nilotinib or imatinib followed by same TKI + PEG‐IFNα 2b	24 months of combination therapy	Not specified

CMR, complete molecular response; DESTINY, De‐Escalation and Stopping Treatment of Imatinib, Nilotinib, or Sprycel in Chronic Myeloid Leukemia; ENEST, Evaluating Nilotinib Efficacy and Safety; EURO‐SKI, European Stop Tyrosine Kinase Inhibitor; IS, International Scale; MMR, major molecular response (*BCR‐ABL1* ^IS^ ≤0.1%); MR^4^, *BCR‐ABL1* ^IS^ ≤0.01%; MR^4.5^, *BCR‐ABL1* ^IS^ ≤0.0032%; PEG‐IFNα, pegylated interferon α; STIM, Stop Imatinib; TFR, treatment‐free remission; TIGER, Tasigna and Interferon Alpha Evaluation Initiated by the German Chronic Myeloid Leukemia Study Group; TKI, tyrosine kinase inhibitor.

Data from all studies to date have shown that TFR can be attempted safely, provided that all pre‐requisites are met and that patients are monitored closely. Molecular relapse was generally not associated with loss of cytogenetic or hematologic responses 8, 12 and patients rapidly regained deep molecular responses after reinitiation of therapy 6, 7, 12, 32. In TWISTER, for example, all patients who reinitiated therapy achieved a second UMRD after a median treatment duration of 3 months 7. Among patients who reinitiated therapy after loss of MMR in A‐STIM, all regained MMR and 23 of 31 regained CMR by the data cutoff (median time to second CMR, 7.3 months) 12. One case of progression to advanced CML following molecular relapse (and after achievement of second MMR following reinitiation of therapy) was reported in A‐STIM 12. Most TFR studies have not reported data addressing whether AEs emerge or resolve following suspension of TKI therapy; however, among 50 patients in a Swedish cohort of EURO‐SKI, 15 (30%) developed musculoskeletal pain within the first 6 weeks after suspending therapy 57. Results from a French cohort multivariate analysis suggest that TKI duration and a medical history of musculoskeletal pain were associated with risk of developing musculoskeletal pain upon suspension of TKI therapy 58. Further investigation is needed to determine the effects of TFR on TKI‐related AEs.

Although the previously discussed studies have established the feasibility of TFR, additional data are needed to determine the optimal criteria and procedures for TFR. Among the criteria that still need to be established are the depth and duration of molecular responses needed before attempting TFR, the appropriate triggers for reinitiating TKI therapy, the sensitivity and frequency of molecular monitoring during a TFR attempt, and patient factors that indicate a high likelihood of successful TFR.

## Defining Sustained Deep Molecular Response and Molecular Relapse

Key aspects of TFR study design relating to the molecular biology of CML, such as the level of response required before attempting TFR and the definition of molecular relapse, differ across studies. If entry criteria are too stringent, some patients who may be able to achieve TFR will be excluded; if they are too lenient, a higher rate of molecular relapse may be observed due to the inclusion of patients with insufficient levels of molecular response 12. Reports of patients who suspended imatinib therapy while in MMR showed that patients rapidly lost MMR and required reinitiation of imatinib 37, 38. Similarly, if the definition of molecular relapse uses a low *BCR‐ABL1* ^IS^ threshold, reinitiation of TKI therapy will be triggered in patients with low but stable levels of *BCR‐ABL1* who may have otherwise been able to maintain TFR successfully 12. The optimal definitions of molecular relapse and triggers for reinitiation of TKI therapy are not yet known; however, data from several trials suggest that loss of MMR is a safe and robust trigger 9, 12, 13, 14. Rates of successful maintenance of TFR in these studies were higher than in those defining molecular relapse as loss of UMRD or loss of CMR 7, 12, 13, 14, 52, although further follow‐up is required to determine whether the trigger to reinitiate therapy affects TFR durability. Many patients maintained persistently low, detectable levels of *BCR‐ABL1* without losing MMR, and patients who lost MMR and reinitiated therapy quickly returned to MMR and CMR 12.

In addition to absolute *BCR‐ABL1* ^IS^ levels, the kinetics of molecular relapse may also be an important factor to consider when determining whether patients should reinitiate therapy. In TWISTER, investigators described two patterns of molecular relapse based on the time required for patient *BCR‐ABL1* transcript levels to double 7. Patients who lost UMRD within the first 6 months after suspension of therapy had short *BCR‐ABL1* doubling times (i.e., *BCR‐ABL1* transcript levels increased rapidly), and the investigators suggested that this group of patients would likely have lost MMR shortly after loss of UMRD. In contrast, patients who lost UMRD >6 months after suspension of therapy had longer *BCR‐ABL1* doubling times (i.e., *BCR‐ABL1* transcript levels increased slowly). The investigators hypothesized that, had therapy not been reinitiated upon confirmed loss of UMRD, *BCR‐ABL1* levels in some of these patients may not have increased dramatically; therefore, loss of MMR may be a more appropriate trigger for reinitiation of therapy in cases of late relapse 7.

## Molecular Monitoring in TFR Studies

Frequent, highly sensitive, standardized molecular monitoring is crucial in TFR studies because molecular relapse can occur after suspension of TKI therapy. Frequent molecular monitoring ensures that these patients will be identified quickly, allowing prompt reinitiation of TKI therapy and achievement of molecular responses 7. TFR clinical trials often call for molecular responses to be monitored monthly for the first 6 or 12 months after suspension of TKI therapy and every 2–3 months thereafter 6, 7, 9, 12, 13, 14, 15, 55. In most TFR studies to date, molecular relapses were monitored in centralized laboratories standardized to the IS 6, 7, 9, 15. In EURO‐SKI, however, molecular responses are being monitored by local standardized laboratories in the EUTOS network 13, 55; results from EURO‐SKI may therefore provide valuable information about the relative importance of standardized versus centralized monitoring. Regardless of the location of molecular monitoring, molecular responses cannot be quantified without the IS 18. When results are reported from TFR studies, precise description of molecular monitoring methods is necessary to ensure that the results can be interpreted properly. With standardized molecular monitoring and well‐defined sensitivity, the findings of a TFR study can be applied to the design of future studies. However, results from studies that use nonstandardized or incompletely described molecular monitoring cannot be readily interpreted and, therefore, cannot be used to inform the design of future studies. TFR study descriptions should include the response level required to attempt TFR, whether centralized and/or standardized laboratories were used, the sensitivity of molecular monitoring, the frequency of molecular monitoring following suspension of therapy, and the evaluation result that triggered reinitiation of therapy.

## Clinical and Biological Factors Associated With Successful TFR

A substantial proportion of participants in TFR trials experience molecular relapse 6, 7, 8, 9, 10, 11, 12, 13, 14, 15, 16, 17. Although the majority of these patients can regain deep molecular responses upon reinitiation of therapy 6, 12, 16, 59, a more complete understanding of which patients are most likely to achieve TFR would lead to stronger eligibility criteria and may help ease patient concerns about attempting TFR 33. Studies have suggested that several patient characteristics seem to be associated with successful TFR, but results have not been consistent between studies (Table 4). A multivariate analysis of data from STIM1 identified low Sokal risk score as an independent predictor of successful TFR 16. In TWISTER, no effect of Sokal risk score was detected, but long (>12 months) duration of interferon therapy prior to imatinib and short (≤9 months) time to achieve UMRD after switching from interferon to imatinib were associated with higher rates of successful TFR 7. In KIDS, factors associated with successful TFR included longer (≥62 months) imatinib duration, presence of imatinib withdrawal syndrome, and negative digital PCR at the time of imatinib cessation 9. In the ISAV study, age (≥45 years) and negative digital PCR at enrollment were associated with successful TFR; no patients <45 years of age with positive digital PCR at enrollment had successful TFR at 24 months 54. In A‐STIM and a Japanese study using similar criteria (JALSG‐STIM213), no significant predictive factors were identified 12, 17. However, as in TWISTER 59, there was a trend (*P* = 0.061) for lower rates of molecular relapse among patients with prior interferon therapy versus those without prior interferon therapy 12. Among patients attempting TFR following nilotinib or dasatinib in STOP 2G‐TKI, a prior history of suboptimal response or resistance to imatinib was significantly (*P* = 0.04) associated with a decreased probability of successful TFR 39.

**Table 4:** Factors associated with successful TFR observed in clinical trials

TFR rates, %^b^	Study (TFR time point)								
	STIM1 16, 52 (median follow‐up, 65 months)	TWISTER 7 (at 3 year)	KIDS^a^, ^9^ (follow‐up ≥1 years)	A‐STIM 12 (at 2 years)	EURO‐SKI 30 (at 6 months)	STOP 2G‐TKI 39 (at 1 years)	DADI^b^ ^15^(at 1 years)	JALSG‐STIM213^b^ ^17^ (at 1 years)	ISAV^d^ ^54^ (median follow‐up, 28.0 month)
Age	Not significant	Above median: 32.8%Below median: 55.0%(*P *>* *0.05)^c^	Not significant	<55.1 year: 69.7%>55.1 years: 57.5%(*P *=* *0.199)	Not evaluated	Not significant	<45 years: 29.4%≥45 years: 54.3%(*P *=* *0.079)	Not significant	Significant^f^
Sex	Not significant	Male: 52.6%Female: 36.7%(*P *>* *0.05)	Male: 55.1%Female: 60.7%(*P *=* *0.424)	Male: 64.3%Female: 63.0%(*P *=* *0.944)	Not evaluated	Not significant	Male: 39.0%Female: 63.6%(*P *=* *0.13)	Not significant	Not evaluated
Sokal score	Low Sokal risk(*P *=* *0.0149)	Low: 51.1%Intermediate: 36.5%High: 25.0%(*P *>* *0.05)	Low: 62.1%Intermediate: 60.6%High: 46.7%(*P *=* *0.300)	Low: 65.9%Intermediate: 63.6%High: 58.1%(*P *=* *0.862)	Not evaluated	Not evaluated	Low: 51.2% (*P *=* *0.68 vs. high)Intermediate: 22.2% (*P *=* *0.27 vs. high)High: 44.4%	Not significant	Not significant
TKI treatment duration	Not significant^f^	Above median: 45.0%Below median: 42.9%(*P *>* *0.05*)* c	<62 month: 40.0%≥62 months: 65.7%(*P *=* *0.013)	<78.7 month: 62.4%>78.7 months: 65.0%(*P *=* *0.788)	<8 years: 53%>8 years: 4%(*P *=* *0.005)	Not significant	<50 months: 35.3%≥50 months: 52.2%(*P *=* *0.12)	Not significant^e^	Not significant
Time to UMRD	Not significant	<244 days after switching from IFN to imatinib: 75.0%>244 days: 33.3%(*P *=* *0.04)	<24 month: 48.6%≥24 months: 64.8%(*P *=* *0.152)	<29.1 months: 59.8%>29.1 months: 67.5%(*P *=* *0.545)	Not applicable	Not evaluated	Not evaluated	Not applicable	Not evaluated
Duration of UMRD prior to discontinuation	Not significant	Above median: 33.7%Below median: 50.0%(*P *>* *0.05*)* c	<36 months: 48.4%≥36 months: 64.8%(*P *=* *0.084)	<41.3 months: 57.5%>41.3 months: 69.6%(*P *=* *0.228)	Not applicable^f^	Not significant	Not evaluated	Not applicable	Not significant
Prior IFN exposure	Not significant	Yes: 51.9%No: 33.7%(*P *>* *0.05)	Not evaluated	Yes: 71.2%No: 55.3%(*P *=* *0.061)	Not evaluated	Not significant	Yes: 53.8%No: 46.0%(*P *=* *0.60)	Not significant	Not significant
2G‐TKI type	Not applicable	Not applicable	Not applicable	Not applicable	Not evaluated	Not significant	Not applicable	Not applicable	Not applicable
Reason for treatment with 2G‐TKI	Not applicable	Not applicable	Not applicable	Not applicable	Not evaluated	SoR/resistance: 41.7%Other: 67.3%(*P *=* *0.04)	Patient's choice: 50.0%Resistance: 7.7%(*P *=* *0.029 vs. patient's choice)Intolerance: 61.1% (*P *=* *0.50 vs. patient's choice)	Not applicable	Not applicable

2G, second‐generation; A‐STIM, According to STIM; DADI, Dasatinib Discontinuation; EURO‐SKI, European Stop Tyrosine Kinase Inhibitor; IFN, interferon; ISAV, Imatinib Suspension and Validation; JALSG, Japanese Adult Leukemia Study Group; KIDS, Korean Imatinib Discontinuation Study; NK, natural killer; SoR, suboptimal response; STIM1, Stop Imatinib 1; TFR, treatment‐free remission; TKI, tyrosine kinase inhibitor; UMRD, undetectable minimal residual disease.

TFR rates were not reported according to each baseline factor.

a Additional baseline characteristics associated with TFR success were digital PCR negativity at screening (positive, 37.5%; negative, 63.8%; *P *=* *0.021) and imatinib withdrawal (yes, 79.5%; no, 49.2%; *P *=* *0.003).

High NK‐cell (CD3‐/CD56^+^ [*P *=* *0.017] and CD16^+^/CD56^+^ [*P *=* *0.0053]) and NK‐cell large granular lymphocyte (CD56^+^/CD57^+^; *P *=* *0.022) counts and low γδ+ T‐cell (*P *=* *0.0022) and CD4^+^ regulatory T‐cell (*P *=* *0.011) counts were also associated with successful TFR.

In JALSG‐STIM213, patients attempting TFR (*n* = 68) were required to have had ≥3 years of imatinib treatment, MR^4^ for >24 month, and MR^4.5^ confirmed at screening. Molecular relapse was defined as loss of MMR.

b Patients ≥45 years of age with negative digital PCR at enrollment had a lower risk of molecular relapse (36.1%) compared with patients <45 years of age with positive digital PCR at enrollment (100%).

c Median values were not reported.

An inverse relationship between age and risk of relapse was observed. Specific rates of TFR according to age were not reported.

d An earlier multivariate regression analysis in STIM1 after a median follow‐up duration of 30 months identified low Sokal risk score (*P* = 0.0009) and ≥60 months of prior imatinib therapy (*P* = 0.0183) as predictive of successful TFR 52.

e A trend for a higher rate of TFR in patients with ≥8 years of imatinib treatment was reported (*P *=* *0.238).

f Rate of TFR at 6 months was not significantly different in patients with a duration of MR^4^ >5 years (68%) versus <5 years (54%; *P *=* *0.07).

Some analyses have identified immunologic factors associated with molecular relapse‐free survival. In DADI, high NK‐cell (CD3−/CD56^+^ [*P *=* *0.017] and CD16^+^/CD56^+^ [*P *=* *0.0053]) and NK‐cell large granular lymphocyte (CD56^+^/CD57^+^; *P *=* *0.022) counts and low γδ+ T‐cell (*P *=* *0.0022) and CD4^+^ regulatory T‐cell (*P *=* *0.011) counts were associated with successful TFR 15. In addition, patients with higher NK‐cell counts at the time of TKI discontinuation were more likely to have successful TFR in separate substudies from STIM1 (*P *=* *0.015) and EURO‐SKI (*P *=* *0.001) 40, 60. In both substudies, the higher NK‐cell count in nonrelapsing patients was due to increased frequencies of mature CD56^dim^ cells relative to CD56^bright^ cells 40, 60. A separate substudy from EURO‐SKI found that patients with lower frequencies of CD86‐positive plasmacytoid dendritic cells had a higher rate of successful TFR (*P *<* *0.001) 41.

## Can We Cure CML?

In the most rigorous sense, curing CML would require complete eradication of CML cells from the patient's body, including leukemic stem cells. This level of cure has remained elusive and may not be necessary because even patients in remission following HSCT can have detectable *BCR‐ABL1* 61, 62. An operational cure, in which patients with minimal levels of residual disease burden might remain in remission without requiring ongoing treatment 63, may be a more appropriate goal. Results from TFR studies to date suggest that some patients with CML with sustained deep molecular responses may be able to achieve an operational cure.

Comparable concepts are being discussed in other diseases, such as breast cancer and human immunodeficiency virus (HIV) infection 64, 65. In rare cases, patients with metastatic breast cancer have been able to maintain complete responses following suspension of therapy 64. Similarly, although the majority of patients with HIV require continued antiretroviral therapy, some are able to maintain a low viral reservoir after stopping therapy, a concept referred to in the HIV field as functional cure 65.

## Conclusions

Although achievement of deep molecular responses (beyond MMR) is not specified as the goal of therapy for CML‐CP in current treatment guidelines, it is associated with multiple meaningful benefits for patients, including improved long‐term clinical outcomes and the potential for TFR. Achievement of a deep molecular response is an appropriate goal for many patients with CML‐CP due to the associated increase in overall survival and avoidance of disease progression 5, 48, 49.

For some patients who achieve sustained deep molecular responses on TKI therapy, TFR is emerging as a feasible goal. Although *BCR‐ABL1* is often detectable in patients in TFR, the ability of these patients to successfully maintain TFR for several years supports the notion that an operational cure may be achievable with TKI therapy. TFR may be appealing to many patients with CML; however, TFR is still investigational, and most patients would likely not attempt TFR if the risk of relapse is high 35. Current NCCN and ELN recommendations suggest that TFR be attempted only in the context of a clinical trial 18, 19. Results from ongoing TFR studies will continue to increase our understanding of the molecular biology of TFR, which may lead to a better ability to predict which patients will be able to successfully maintain TFR.

## Conflict of Interest

Stéphanie Dulucq has no conflicts of interest to disclose. François‐Xavier Mahon received honoraria from Novartis Pharmaceuticals Corporation, Bristol‐Myers Squibb, Ariad, and Pfizer; acted as a member on an advisory committee with Novartis Pharmaceuticals Corporation, Bristol‐Myers Squibb, and Ariad; and received research funding from Novartis Pharmaceuticals Corporation.
